# Intelligent Predictor of Energy Expenditure with the Use of Patch-Type Sensor Module

**DOI:** 10.3390/s121114382

**Published:** 2012-10-25

**Authors:** Meina Li, Keun-Chang Kwak, Youn-Tae Kim

**Affiliations:** 1 Department of IT Fusion Technology, Graduate School, Chosun University, 375 Seosuk-dong, Gwangju 501-759, Korea; E-Mail: limeinaj1@hotmail.com; 2 Department of Control, Instrumentation, and Robot Engineering, Chosun University, 375 Seosuk-dong, Gwangju 501-759, Korea; E-Mail: kwak@chosun.ac.kr

**Keywords:** intelligent predictor, energy expenditure, patch-type sensor module, heart rate, movement index, linguistic model

## Abstract

This paper is concerned with an intelligent predictor of energy expenditure (EE) using a developed patch-type sensor module for wireless monitoring of heart rate (HR) and movement index (MI). For this purpose, an intelligent predictor is designed by an advanced linguistic model (LM) with interval prediction based on fuzzy granulation that can be realized by context-based fuzzy c-means (CFCM) clustering. The system components consist of a sensor board, the rubber case, and the communication module with built-in analysis algorithm. This sensor is patched onto the user's chest to obtain physiological data in indoor and outdoor environments. The prediction performance was demonstrated by root mean square error (RMSE). The prediction performance was obtained as the number of contexts and clusters increased from 2 to 6, respectively. Thirty participants were recruited from Chosun University to take part in this study. The data sets were recorded during normal walking, brisk walking, slow running, and jogging in an outdoor environment and treadmill running in an indoor environment, respectively. We randomly divided the data set into training (60%) and test data set (40%) in the normalized space during 10 iterations. The training data set is used for model construction, while the test set is used for model validation. The experimental results revealed that the prediction error on treadmill running simulation was improved by about 51% and 12% in comparison to conventional LM for training and checking data set, respectively.

## 1. Introduction

Energy expenditure (EE) refers to the amount of energy that a person uses daily to complete all bodily activities from movement to breathing. The accurate measurement of EE from physical activity is a challenging problem that is important to epidemiologists, exercise scientists, clinicians, and behavioral researchers. Recently the need for wireless health monitoring and detection of emergency situations has rapidly increased [1]. Such a wireless system is used extensively, not only to estimate EE, but it is also useful for patient monitoring and athletic training. Previous commercial physiological sensors are relatively large and their power consumption is also considerable amount, so we recently developed a patch-type sensor module to solve these problems [2]. The leading characteristics of this sensor module are small size, wireless operation, low cost, light weight, and comfort for long-term wear, as well as integration of all required measurement parameters into a single sensor. Furthermore, a communication module is also integrated into the device to transmit heart rate and acceleration information. Oxygen exchange (VO_2_) is one of the most fundamental and widely recognized measures of EE. In general, the measurement of VO_2_ has been confined to laboratory settings and the use of a treadmill due to the sophisticated equipment required. The recent introduction of the Cosmed K4b^2^ portable metabolic analyzer allows measurement of VO_2_ outside of a laboratory setting in more typical clinical or household environments [3]. This analyzer provides measurements of VO_2_ and VCO_2_ during steady-state, submaximal exercise similar to the traditional gas exchange system [4]. The obtained VO_2_ is transformed into EE.

There are many portable systems available. although perhaps none integrated with wireless connectivity. Wong [5] have developed and tested a portable device that measures energy expenditure per unit time of a human subject. Wixted [6] has analyzed the variance of accelerometer-count based energy estimates and identified mechanical, biomechanical, and anthropometrical influences. There are numerous portable instrumentations to measure movement [7–9]. Various methods have demonstrated success in classification or prediction applications [10–15]. Oliver [11] has investigated the utility of a variety of active accelerometer count thresholds for classifying sitting time in a sample of office workers. Xiao [12] has designed a prediction model using the feedforward neural network (FFNN) to reflect the effects of physical activities on the heart rate. In [13], a multi-step HR prediction method is proposed. The HR prediction problem was converted into an initial-value problem for ordinary differential equations. Then the Adams-Bashforth method was used to implement a multi-step prediction. Yuchi [14] and Xiao [15] proposed the well-known FFNN as the predictor model based on the relationship between heart rate and physical activity. The FFNN experimental results showed the potential of the predictor with the results close to the actual data. Vathsangam [23] estimated energy expenditure during treadmill walking using a single hip-mounted inertial sensor with a triaxial accelerometer and triaxial gyroscope. He performed a comparative analysis of the well-known probabilistic techniques in conjunction with inertial data modeling to predict energy expenditure for steady-state treadmill walking. Nonlinear regression methods showed better prediction accuracy compared to linear methods. Lin [24] presented a wearable sensor module and two representative neural networks (RBFN, GRNN) for activity classification and prediction. He demonstrated the effectiveness of a wearable sensor module and its neural network-based activity classification algorithm for energy expenditure. The research in the literature mentioned above has been performed based on well-known neural networks [12–15,24] or statistical methods [23] from numerical data. However, these methods have not been considered to be knowledge representation via fuzzy if-then rules with meaningful linguistic labels. In general, it is frequently advantageous to use several computing techniques synergistically rather than exclusively, resulting in construction of complementary hybrid intelligent systems such as neural-fuzzy computing. The effectiveness of these complementary approaches has been demonstrated [21]. Therefore, our method shall be developed to possess intensive computational ability, together with meaningful linguistic labels [16].

This paper focuses on a method for designing an intelligent predictor of EE using the developed patch-type sensor module for wireless monitoring of a given input-output data such as heart rate (HR), movement index (MI), and EE. It has been demonstrated that the device used in this paper is suitable through reliability tests, performance evaluation of heart rate detection and movement indexes, as well as field tests using the Bruce protocol [2]. The intelligent predictor is designed by a Takagi-Sugeno-Kang-Linguistic Model (TSK-LM) with an interval prediction with the aid of fuzzy granulation realized via Context-based Fuzzy c-Means (CFCM) clustering [16]. This clustering technique builds information granules in the form of fuzzy sets and develops clusters by preserving the homogeneity of the clustered patterns associated with the input and output space [17–21]. The conventional LM was designed by linguistic contexts in the consequent part [19]. Although these contexts give meaningful linguistic labels, the obtained results did not show a good performance. The TSK type is by far the most popular candidate for fuzzy modeling and effective to develop a systematic approach [21]. Based on these two complementary approaches, we propose a TSK-based linguistic type in the subsequent work. The normal walking, brisk walking, slow running, and jogging experiments were performed in outdoor environments, as well as treadmill running in indoor environments [3]. The experiments results revealed that the proposed intelligent predictor outperformed the well-known methods [18,19,22]. The experimental results revealed that the prediction error on treadmill running simulation was improved by about 49∼51% and 12% in comparison to RBFN-CFCM and conventional LM for training and checking data set, respectively. We also obtained 70% and 14% performance improvement in comparison to conventional RBFN for training and checking data set, respectively. In the case of outdoor environments, we obtained about 28∼39% and 4∼8% improvement in comparison to conventional LM for training and checking data set, respectively.

The material of this paper is organized in the following fashion: in Section 2, we describe a patch-type sensor module to obtain the HR and MI. In Section 3, we present the intelligent predictor based on TSK-LM from numerical input-output physiological data pairs. In Section 4, we report the experimental setup and perform a comparison in indoor environments. Finally the conclusions and comments are given in Section 5.

## 2. Wireless Monitoring System with a Patch-Type Sensor Module

**Figure 1.**
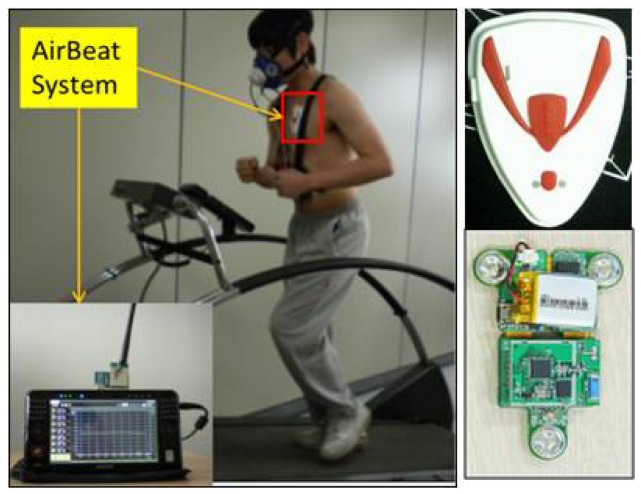
Patch-type sensor module and Cosmed K4b^2^ gas system during treadmill running.

This section covers the details of the patch-type sensor module for wireless monitoring of heart rate and movement index. This module consists of a sensor board, rubber board, and communication module. Figure 1 shows the module equipped with sensor board and silicon packaging case during treadmill running in an indoor environment [2].

**Figure 2.**
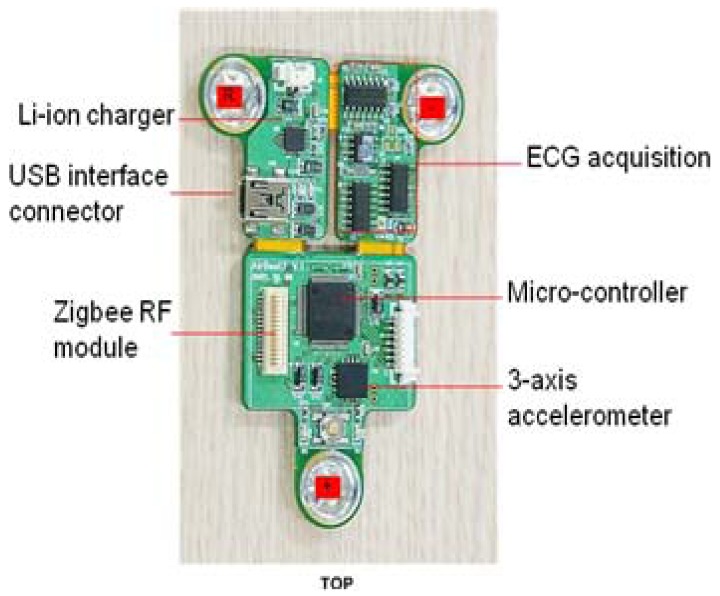
Patch-type sensor board with 3-axis accelerometer and ECG acquisition.

The patch-type sensor board includes a Li-ion charger, USB interface connector, Zigbee RF module, ECG acquisition, micro-controller, voltage regulators, voltage converter, and 3-axis accelerometer. Here a Texas Instruments MSP 430 chip with 60 KB flash memory, 2048 SRAM memory, and 8 MHz clock frequency is used in the microcontroller. The input signals are interpreted appropriately as heart rate, heat stress, and movement index using a signal processing algorithm. Simultaneous, dependable real-time communication has been secured for a distance of over 400 m for eight people. To enable wireless exercise management, the acquired sensor data should be passed to a central monitoring system. In this system, a commercial Zigbee telecommunication module is used for data transfer [2]. Figure 2 shows the patch-type sensor board with 3-axis accelerometer and ECG acquisition [2].

In the heart rate monitor design, we used three electrodes for ECG measurement on the chest. These gel-free electrodes are mounted on a conductive adhesive patch. The ECG analog circuitry was specifically designed for effective motion artifact rejection during exercise. The ECG signal is converted into a digital signal with a sampling rate of 200 Hz for heart rate estimation. The performance evaluation of the heart rate detection algorithm was conducted by comparing it with the reference system, while changing the running speed. The average error between the heart rate monitor and the commercial stress ECG monitor was less than 2%.

**Figure 3.**
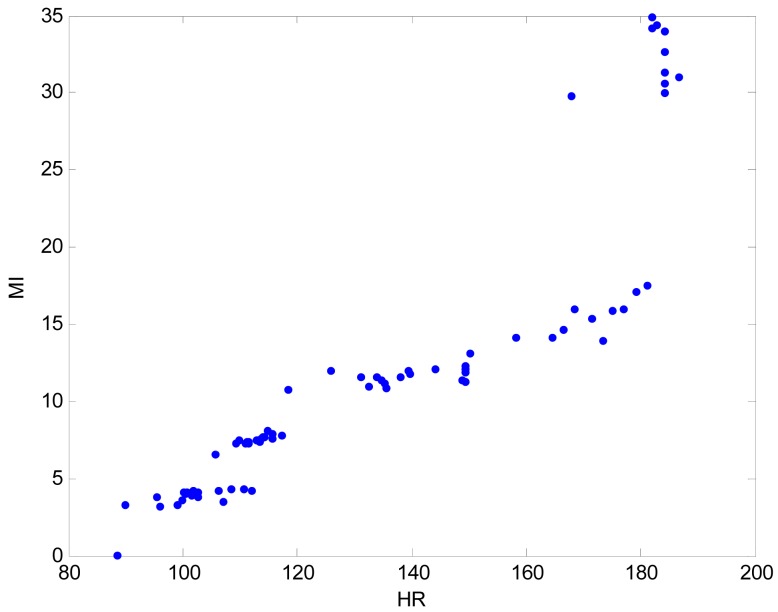
Example of data distribution during treadmill running.

We used a three-axis accelerometer (MMA7260Q) to detect acceleration changes during exercise. This device is a cheap capacitive micro-machined accelerometer featuring good sensitivity, low power consumption, and very small size. With this sensor, this system can measure the athlete's motion signals in the range of -6 to 6 g and calculate the movement index. We used the zero-crossing detection algorithm for the motion artifact rejection. This procedure reduces the probability of false-value extraction due to motion artifact noises [2]. The performance evaluation of the movement index was conducted on a feature-point extraction function. It showed good feature-point extraction characteristics with a maximum of 1%. Figure 3 shows data distribution of heart rate and movement index during treadmill running. The pairwise linear correlation coefficient between HR and MI is 0.892.

**Figure 4.**
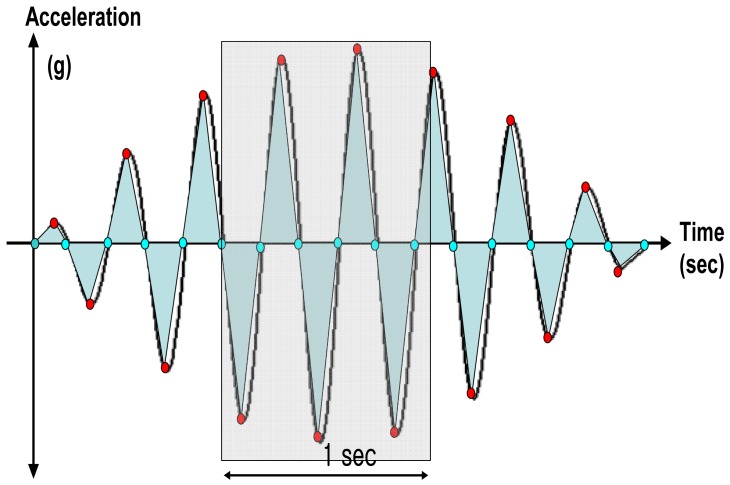
Acceleration waveform and the method of defining the movement index (the sum of areas per second) [2].

Figure 4 visualizes theoretical acceleration waveform with feature points, feature values, and movement index. The movement index can be defined as the average of the triangular areas of the acceleration graph for each second in the acceleration waveform as shown in Figure 4. For further details on the theoretical acceleration waveforms, readers may refer to [2].

## 3. Intelligent Predictor by Linguistic Model

**Figure 5.**
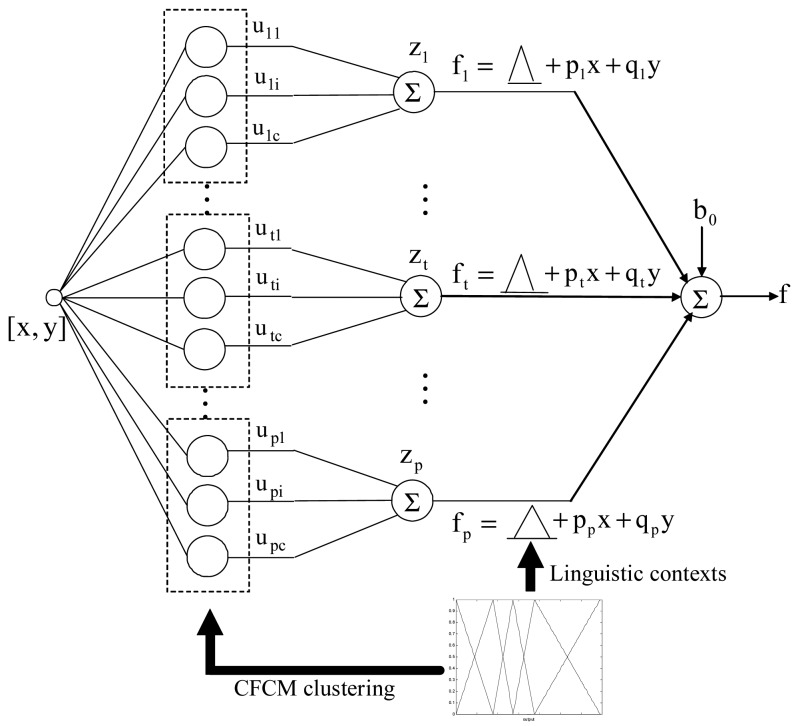
Architecture of TSK-LM as intelligence predictor.

For simplicity, we assume that the TSK-LM under consideration has two inputs x and y. Moreover, we assume that the number of the cluster centers in each context is equal. The output of this model is produced by the lower and upper bound with uncertainty because the estimated output is computed by the fuzzy number. The TSK-LM architecture is shown in Figure 5.

Every circle node of the second layer represents the membership grade of the fuzzy set associated with a linguistic label. Here fuzzy clustering in each linguistic context is performed in dashed-line rectangular area. Every node in the third layer is a fixed node labeled *Σ*, which computes the summation of overall membership grades obtained by each context. The *zt*, *t* = *1*, *2*, …, *p*, is the summation of membership values corresponding to the *t*-th context. The single node in the fourth layer is also a fixed node which computes the overall output as the summation of all incoming signals.

The linguistic contexts are used to extract fuzzy rules in the CFCM clustering. In the conventional LM, these contexts were generated through a series of triangular membership functions equally spaced along the domain of an output variable. However, we may encounter a data scarcity problem due to small data sets included in some linguistic contexts. Thus, this problem brings about the difficulty to obtain fuzzy rules from the Context-based Fuzzy c-Means (CFCM) clustering [17]. Therefore, we use the probability distribution of output variable to produce the flexible linguistic contexts.

**Figure 6.**
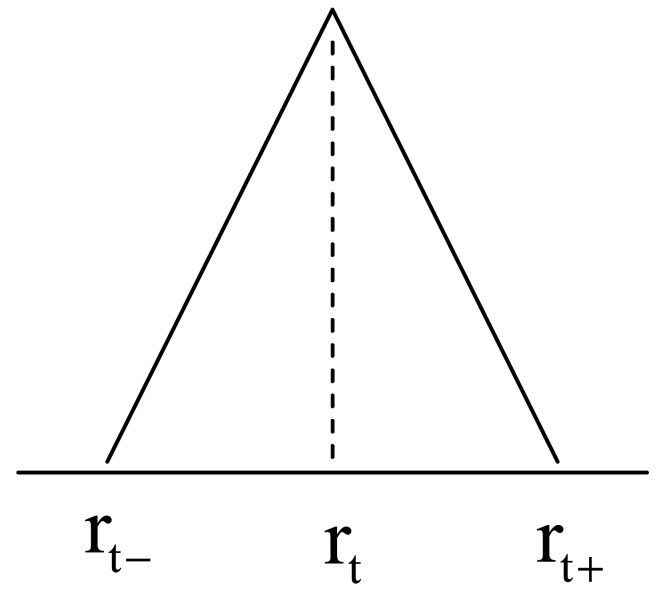
The t-th linguistic context in consequent part.

The CFCM clustering method, as proposed by Pedrycz [17], is an effective approach to estimate the cluster centers preserving homogeneity on the basis of fuzzy granulation. In contrast to the context-free clustering methods, the CFCM clustering method is performed with the aid of the information granulation. As shown in Figure 5, CFCM clustering is performed in the second layer. Here the membership matrix is initialized with random values between 0 and 1. The optimization completed by the CFCM clustering is realized iteratively by updating the membership matrix and the cluster centers. The update of the membership matrix is completed as follows:

(1) $$\begin{array}{ccc} u_{\text{tik}}=\frac{w_{t_{k}}}{{\sum_{j=1}^{c}\left(\frac{\left\|x_{k}-v_{i}\right\|}{\left\|x_{k}-v_{j}\right\|}\right)}^{\frac{2}{m-1}}} & i=1,2,\ldots ,c, & k=1,2,\ldots ,N \end{array}$$

where *u_tik_* represents the element of the membership matrix induced by the *i*-th cluster and *k*-th data in the *t*-th context. Here *w_tk_* denotes a membership value of the *k*-th data to the *t*-th context. The cluster centers *v_i_* are calculated in the form:

(2) $$v_{i}=\frac{\sum_{k=1}^{N}u_{\text{tik}}^{m}x_{k}}{\sum_{k=1}^{N}u_{\text{tik}}^{m}}$$

where the fuzzification factor “*m*” is taken as 2.0. When applying the CFCM clustering to numerical input-output data pairs, each of the cluster centers presents a prototype that exhibits certain characteristics of the system to be modeled. The *t*-th linguistic context is visualized in Figure 6, where [*r_t_*_−_, *r_t_*, *r_t_*_+_] is a 3-element vector that determines the break points of this membership function. Thus the *t*-th consequent part combined with TSK-type is expressed as follows:

(3) $$f_{t}=[r_{t-},r_{t},r_{t+}]+p_{t}x+q_{t}y$$

where *f_t_* is a vector represented by [*f_t_*_−_, f_t__*_, *f_t_*_+_]. Here the parameters of linguistic contexts are obtained by probabilistic distribution. The linear coefficients {*p_t_*, *q_t_*} of TSK-type are estimated by Least Square Estimator (LSE).

The results obtained by conventional LM have shown a biased prediction error. This problem brings about a poor approximation and generalization ability. Therefore, we add a bias term to the conventional LM so that the TSK-LM can provide an unbiased prediction. The bias term is computed in a straightforward manner as follows:

(4) $$b_{0}=\frac{1}{N}\sum_{k=1}^{N}\left({\text{target}}_{k}-{\text{predict}}_{k}\right)$$

where predict*_k_* denotes a modal value of fuzzy number produced for *k*-th input data point and *N* is the number of data point. The resulting fuzzy number with bias term is expressed as the following form:

(5) $$f=z_{1}⊗f_{1}⊕\cdots ⊕z_{p}⊗f_{p}⊕\cdots ⊕z_{t}f_{t}+b_{0}$$

We denote the algebraic operations by ⊗ and ⊕ to emphasize that the underlying computation operates on a collection of fuzzy numbers. Given the multiplication and addition for two operations, the final fuzzy number (model output) is computed as follows:]

(6) $$f_{*}=\sum_{t=1}^{p}z_{t}f_{t*}+b_{0}=\sum_{t=1}^{p}z_{t}(r_{t}+p_{t}x+q_{t}y)+b_{0}$$

Furthermore, the lower and upper bound of model output are computed by the following form [4]:

(7) $$f_{-}=\sum_{t=1}^{p}z_{t}(r_{t-}+p_{t}x+q_{t}y)+b_{0}$$

(8) $$f_{+}=\sum_{t=1}^{p}z_{t}(r_{t+}+p_{t}x+q_{t}y)+b_{0}$$

Based on these bounds, we can represent the uncertain model output with fuzzy number.

## 4. Experimental Results

**Figure 7.**
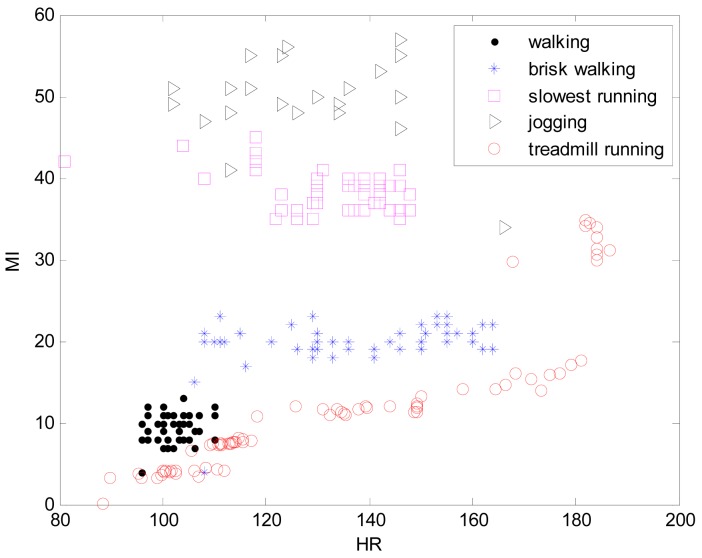
Distribution of two input variables obtained in indoor and outdoor environments.

The system presented in this paper is a wireless, small size (6 cm × 9 cm) and light weight (41 g) sensor that can be patched on the chest of participants to obtain physiological data as shown in Figure 1. The sensor board consists of a 3-axis accelerometer and three ECG electrodes to detect heart rate (HR) and movement index (MI). This system can simultaneously monitor a maximum of eight participants in an open field over 400 meters. The Cosmed K4b^2^ portable metabolic system is used as the standard value to estimate energy expenditure (EE). Thirty participants (n = 30) were recruited from Chosun University to take part in this study. The participants were advised of this study through a University bulletin. All of the recruited participants reported no chronic diseases or activity disabilities. Data regarding age, height, and weight were collected by survey. The body mass index (BMI) was calculated using weight (kg) per squared height (m^2^). Table 1 lists mean and standard deviation regarding the physical characteristics of the subjects. The participants were shown how to complete the procedure and understood the schedule based on a written paper before starting the experiment. Approval from the Ethics Committee of the Chosun University Medical Centre was obtained before beginning the study. The experiments were performed in both indoor and outdoor environments. Figure 7 visualizes the distribution of two input variables obtained from normal walking, brisk walking, slow running, and jogging in an outdoor environment and treadmill running in an indoor environment, respectively.

Firstly, we performed a treadmill running test in an indoor environment. The participants are tested on the treadmill based on the submaximal protocols. The treadmill is started at 2.7 km/h with 10% increase. The treadmill slope increases by 2% every three minutes. The database is recorded by the system in real-time around 9∼12 minutes. The EE is simultaneously measured by the portable indirect calorimeter. In this experiment, we shall apply the TSK-LM for the prediction problem of EE as nonlinear regression. The EE depends on two continuous attributes, including HR and MI. The data set consists of 76 examples. The training and checking data set are randomly selected by 60%–40% split in the normalized space between 0 and 1, respectively. Ten iterations of the experiment were performed. The training data set is used for construction of the intelligent predictor model, while the checking data set is used for intelligent predictor model validation. Thus, the resultant predictor is not biased toward the training data set and it is likely to have a better generalization capacity for new data. The prediction performance by root mean square error (RMSE) is obtained as the number of context and cluster increased from 2 to 6, respectively. Table 2 shows a comparison of RMSE with the previous prediction works. As listed in Table 2, the TSK-LM predictor showed good performance in comparison with the neural networks and LM. The Multilayer Perceptron (MLP) used in Table 2 was designed by logistic activation function, back propagation algorithm, and 2-10-1 network, corresponding to the number of nodes in each layer. Here we used 1,000 epochs and a learning rate of 0.01. In the design of the LM, we used three contexts and three clusters in each context, determined by trial and error. Although the LM has a structured knowledge representation in the form of fuzzy if-then rules, it lacked the adaptability to deal with nonlinear model. Moreover, we performed the experiments as the number of nodes in hidden layer increases from 3 to 20 in the design of Radial Basis Function Networks (RBFN). Finally, we determined the 20 nodes representing best performance.

**Figure 8.**
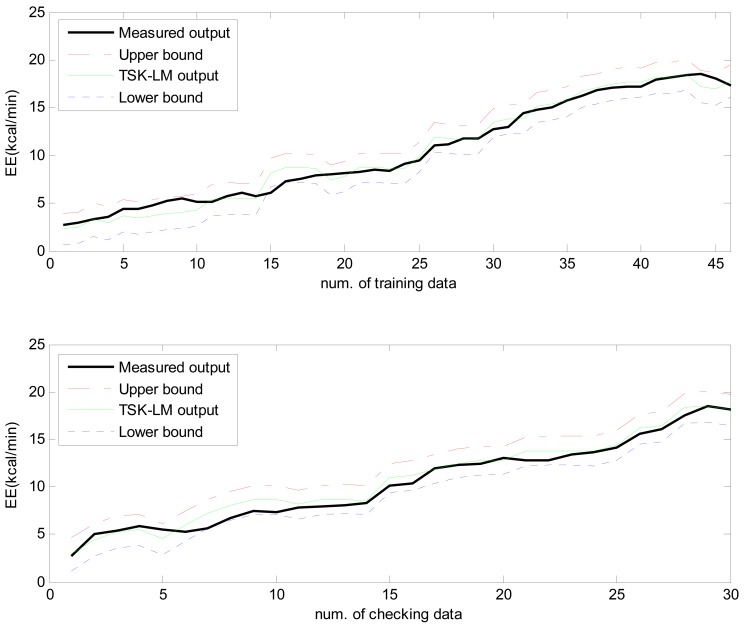
Approximation and generalization capability with interval prediction for training and checking data.

**Figure 9.**
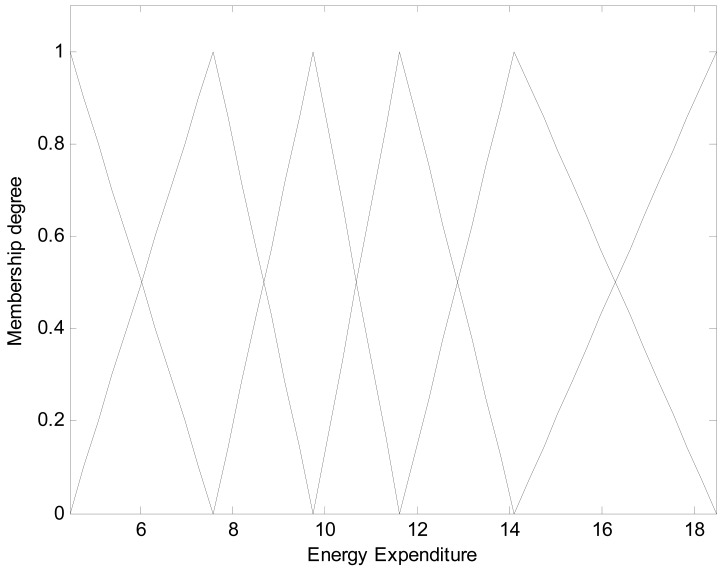
Linguistic contexts produced in output space (p = 6).

**Figure 10.**
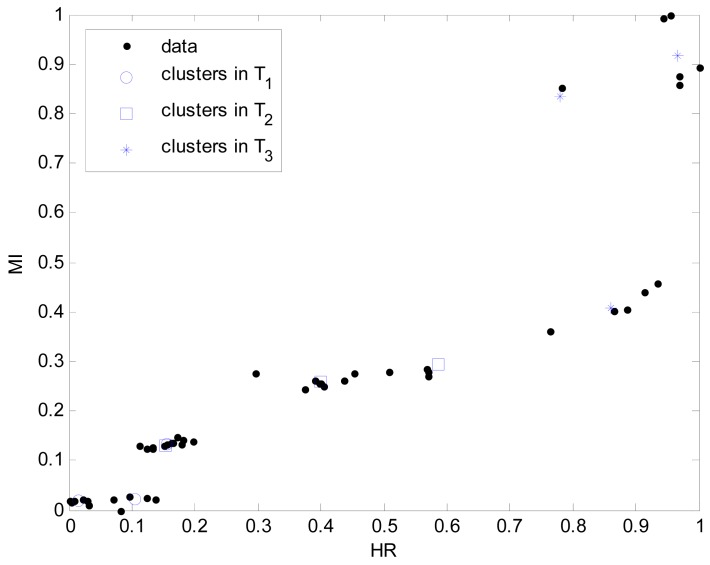
Data distribution and cluster centers estimated in each context.

Here the weights of RBFN were obtained by the least square estimate (LSE) methods. The best TSK-LM model was obtained when the checking error (Chk_RMSE) is minimal (p = 6, c = 3). Figure 8 shows the approximation and generalization capability with an interval prediction by lower and upper bound for training and checking data, respectively. As shown in Figure 8 and Table 2, the experimental results revealed that the presented intelligent predictor (TSK-LM) outperformed the well-known methods [18,19,22]. We obtained about 51% and 12% performance improvement in comparison to LM itself for the training and checking data set, respectively. Figure 9 shows linguistic contexts produced in the EE output space. Figure 10 visualizes the distribution of input variables and cluster centers estimated in each context when p = 3 in the normalized space.

Table 3 lists the factor influence on the prediction performance for the training and checking data sets. The experimental methods and the number of rules are the same as mentioned above. As shown in Table 3, the HR has a strong influence in comparison to MI in the design by LM and TSK-LM. As listed in Table 3, the experimental results of TSK-LM on HR influence showed 73% and 27% improvement in comparison to LM itself for the training and checking data sets, respectively.

**Figure 11.**
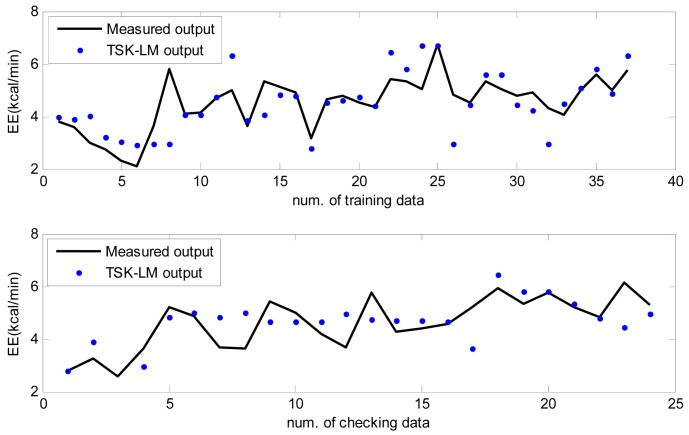
Approximation and generalization capability for normal walking data.

**Figure 14.**
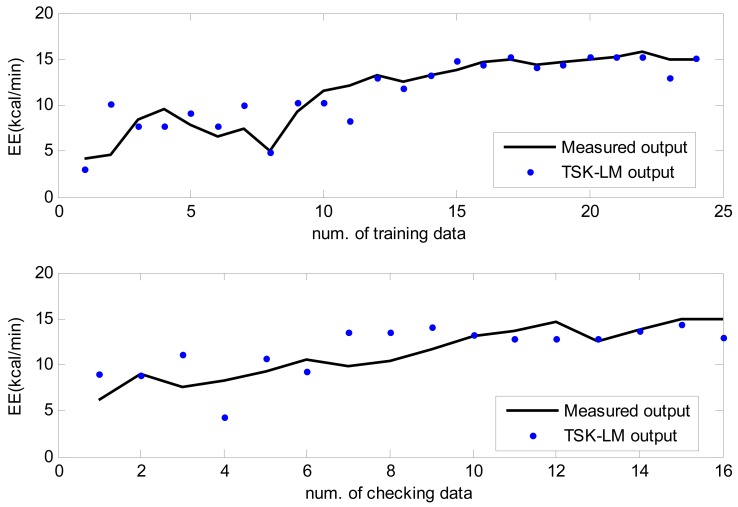
Approximation and generalization capability for jogging data.

We also performed normal walking, brisk walking, slow running, and jogging in an outdoor environment, respectively. All of the participants were encouraged to complete four exercise tests in the open field. Each test course was performed on a 400 meter oval track. The experimental procedure was designed to progress as naturally as possible. The participants were told to be in a pleasurable mood, as in doing exercise in the morning or taking a walk after dinner and to be comfortable when walking and jogging. The approximation and generalization performance are shown in Figure 11 to Figure 14 for normal walking, brisk walking, slow walking, and jogging data, respectively. Table 4 lists performance comparison of RMSE under outdoor environments. In the case of the training data set, we obtained 39%, 29%, 36%, and 28% performance improvement in comparison to conventional LM for normal walking, brisk walking, slow walking, and jogging, respectively. We also achieved about 4∼8% improvement for the checking data set.

## 5. Conclusions

We developed an intelligent TSK-LM predictor of energy expenditure with the aid of a patch-type sensor module for wireless monitoring of heart rate and movement index. This predictor possesses intensive computation ability together with meaningful linguistic labels and interval prediction based on fuzzy granulation. The experimental results revealed that the presented intelligent predictor showed good performance in comparison with the systems described in the previous works. Moreover, we could recognize that the system equipped with patch-type sensor module can be used as an efficient tool to predict energy expenditure for athletic training.

## Figures and Tables

**Figure 12.**
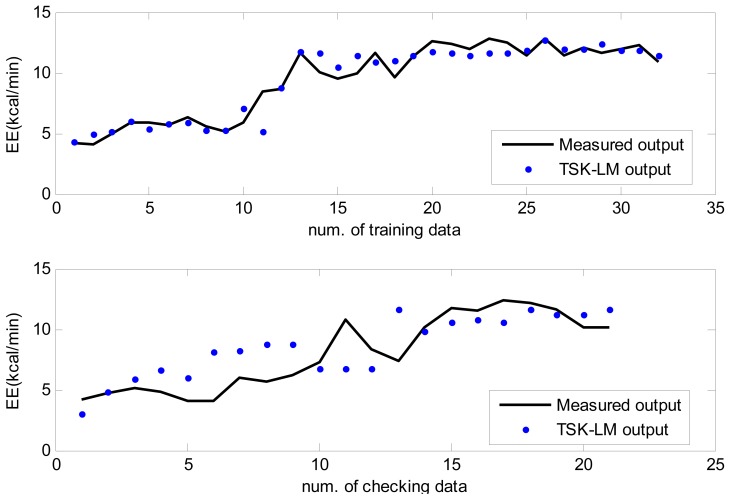
Approximation and generalization capability for brisk walking data.

**Figure 13.**
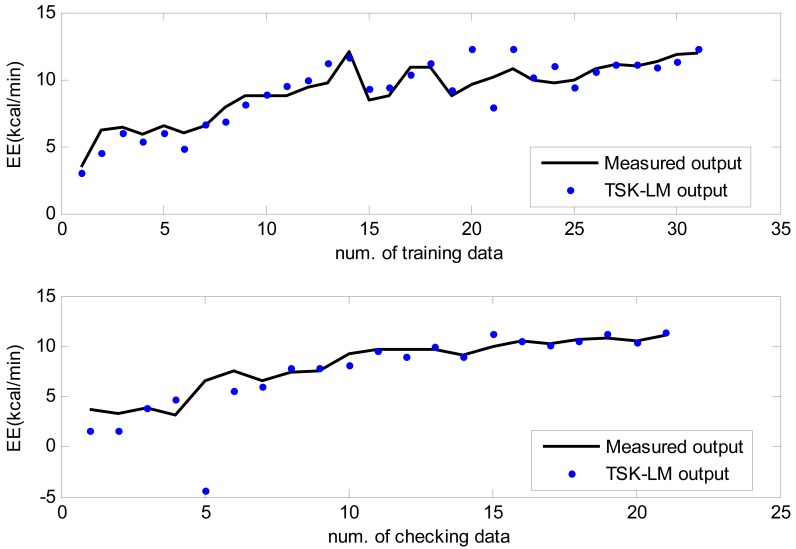
Approximation and generalization capability for slow running data.

**Table 1.** Physical characteristics of subjects.

**Characteristic**	**Men (n = 17)**	**Women (n = 13)**
Age (y)	26.0 ± 2.1	25.8 ± 3.2
Height (cm)	169 ± 6.7	162.1 ± 6.3
Weight (kg)	65.2 ± 9.6	52.1 ± 9.4
BMI (kg.m^−2^)	22.8 ± 7.1	19.8 ± 7.1

**Table 2.** Performance comparison of RMSE (indoor).

	**Trn_RMSE**	**Chk_RMSE**
MLP [22]	1.41	1.42
RBFN [22]	0.73	0.97
RBFN-CFCM [18]	0.64	0.95
LM(p = 3,c = 3) [19]	0.65	0.95
TSK-LM(p = 6,c = 2)	0.52	0.85
TSK-LM(p = 6,c = 3)	0.43	0.85

**Table 3.** Factor influence on prediction performance.

**Factor**	**Method**	**Trn_RMSE**	**Chk_RMSE**
HR	LM [19]	0.97	1.08
	TSK-LM	0.56	0.85
MI	LM [19]	1.35	1.51
	TSK-LM	0.62	1.29

**Table 4.** Performance comparison of RMSE (outdoor).

**Physical activity**	**Method**	**Trn_RMSE**	**Chk_RMSE**

Normal Walking	LM [19]	0.75	1.06
	TSK-LM	0.54	0.98

Brisk Walking	LM [19]	1.47	1.82
	TSK-LM	1.14	1.72

Slow Running	LM [19]	0.79	1.44
	TSK-LM	0.58	1.38

Jogging	LM [19]	2.0	2.74
	TSK-LM	1.56	2.58
